# Prevalence of karyotype alterations in couples with recurrent pregnancy loss in a tertiary center in Brazil

**DOI:** 10.61622/rbgo/2024rbgo51

**Published:** 2024-06-27

**Authors:** Elaine Cristina Fontes de Oliveira, Ines Katerina Damasceno Cavallo Cruzeiro, Cezar Antônio Abreu de Souza, Fernando Marcos Reis

**Affiliations:** 1 Hospital das Clínicas Universidade Federal de Minas Gerais Belo Horizonte MG Brazil Hospital das Clínicas, Universidade Federal de Minas Gerais, Belo Horizonte, MG, Brazil.

**Keywords:** Abortion habitual, Abortion, spontaneous, Chromosome aberrations, Karyotype, Translocation genetic

## Abstract

**Objective:**

To assess the prevalence and type of chromosomal abnormalities in Brazilian couples with recurrent pregnancy loss (RPL) and compare the clinical characteristics of couples with and without chromosome abnormalities.

**Methods:**

We assessed the medical records of 127 couples with a history of two or more miscarriages, referred to a tertiary academic hospital in Belo Horizonte, Brazil, from January 2014 to May 2023. Karyotype was generated from peripheral blood lymphocyte cultures, and cytogenetic analysis was performed according to standard protocols by heat-denatured Giemsa (RHG) banding.

**Results:**

Abnormal karyotypes were detected in 10 couples (7.8%). The prevalence of chromosomal abnormalities was higher among females (6.3%) compared to males (2.0%), but this difference was not statistically significant (p=0.192). The mean number of miscarriages was. 3.3 ± 1.1 in couples with chromosome abnormalities and 3.1 ± 1.5 in couples without chromosome abnormalities (p=0.681). Numerical chromosomal anomalies (6 cases) were more frequent than structural anomalies. Four women presented low-grade Turner mosaicism. No differences were found between couples with and without karyotype alterations, except for maternal age, which was higher in the group with chromosome alterations.

**Conclusion:**

The prevalence of parental chromosomal alterations in our study was higher than in most series described in the literature and was associated with increased maternal age. These findings suggest that karyotyping should be part of the investigation for Brazilian couples with RPL, as identifying the genetic etiology may have implications for subsequent pregnancies.

## Introduction

Recurrent pregnancy loss (RPL) is classically defined as three or more consecutive miscarriages before 20 weeks of gestation.^(1)^ It is a clinical condition that affects around 1% of the couples trying to conceive, but the prevalence rises to 3% if two consecutive miscarriages are considered sufficient for the diagnosis.^(2)^

RPL has been associated with genetic or chromosomal abnormalities in the couple or in the embryo, maternal thrombophilia, uterine structural abnormalities, maternal immune diseases, endocrine disorders, and environmental factors.^(3-5)^

Genetic causes of RPL include specific genetic diseases and chromosomal alterations. These can be investigated in the couple or in the conceptus, accounting for 70% of early miscarriages, especially in advanced maternal age.^(6,7)^ Maternal age is a well-known risk factor for sporadic miscarriage, as well as for RPL. Women over the age of 35 have an increased rate of meiotic errors in oocyte development leading to increased embryonic aneuploidy.^(8)^ Despite being the main diagnosed cause of miscarriage, the occurrence of chromosomal alterations can be random.

Embryonic chromosomal abnormalities may occur during embryo development, or come from parental abnormal ovum or sperm.^(9)^ The parental chromosomal aberration might be either a structural anomaly, such as reciprocal or Robertsonian translocations, or mosaicism for numeric aberrations.^(6,8)^ Because unbalanced gametes generated by chromosomal aberrance are associated with RPL,^(10,11)^the guidelines of some professional societies like the American Society for Reprodutive Medicine (ASRM) suggest the inclusion of parental karyotyping in the investigation of couples with a history of RPL.^(12-14)^

Previous studies have shown that the incidence of chromosomal abnormalities is less than 1% in the general population and 2–5% in couples with RPL.^(15,16)^ Furthermore, around 12% of couples may exhibit structural rearrangements of their chromosomes, with only 40% of these being identified by the traditional karyotypes.^(17)^ Balanced translocation is the most common structural aberration, accounting for 38 % of abnormalities in a retrospective populational study.^(18)^ The second most prevalent anomaly was chromosomal inversion.^(6,18)^

Even though the major cause of miscarriage is conceptus chromosomal abnormalities, some RPL cases have been related to parental karyotype alterations, whose prevalence can vary depending on the population studied. There is a lack of information about these events in Brazilian couples. Thus, the aim of this study was to investigate the prevalence and types of chromosomal abnormalities found in Brazilian couples with RPL referred to a tertiary academic hospital and to compare the clinical characteristics of couples with or without chromosome abnormalities.

## Methods

We searched through the medical records of 127 couples with a history of two or more miscarriages, referred to a tertiary academic hospital in Belo Horizonte, Brazil, from January 2014 to May 2023. RPL was defined as a history of at least two clinical pregnancies (i.e. confirmed by ultrasound and/or clinical examination), interrupted spontaneously before 20 weeks gestation. All patients undertook the following screening tests: couple’s karyotype, lupus anticoagulant antibody, anticardiolipin antibodies (IgM and IgG), anti-b2 glycoprotein 1, serum thyroid stimulating hormone (TSH), serum prolactin (if clinical suspicion of hyperprolactinemia), transvaginal ultrasound, and hysteroscopy.

Karyotype was generated from the peripheral blood lymphocyte cultures during 72 h and cytogenetic analysis was performed according to standard protocols by heat-denatured Giemsa (RHG) banding. Fifteen metaphases were systematically studied, and if any mosaicism was suspected, the number of analyzed metaphases was enlarged to 50. Chromosomal abnormalities were reported in accordance with the current International Standard Cytogenetic Nomenclature (ISCN), an international system for human cytogenomic nomenclature.

According to previous studies,^(19)^ a minimum sample size of 196 couples with a history of RPL is required to estimate the prevalence of chromosomal abnormalities with a significance level of 95% and a margin of error = 5%. Pos-hoc calculation showed that our sample (n=127) can estimate the same prevalence with a margin of error = 8%. Continuous data were reported as mean ± SD and range (minimum-maximum). The categorical variables were described as percentages and the chi-square test or Fisher ´s exact test was performed. Two-sided p<0.05 was considered statistically significant.

This study was approved by the Research Ethics Committee of Universidade Federal de Minas Gerais 5.464.119 (protocol 58024219.3.0000.5149).

## Results

The mean age of female partners was 33.2 ± 5.7 years (range: 21–46 years), whereas the mean age of male partners was 35.3 ± 7.3 years (range: 19–62 years). The number of previous abortions varied from 2 to 11 abortions/couple with a mean of 3.1 ± 1.5. The clinical characteristics of participants are shown in chart 1.

**Chart 1:** Clinical characteristics of the study participants

Characteristics	
Female age, mean ± SD (range)	33.2 ± 5.7 (21-46)
Male age, mean ± SD (range)	35.3 ± 7.3 (19-62)
Number of miscarriages, mean ± SD (range)	3.1 ± 1.5 (2-11)
Number of miscarriages (frequency)	
2	54
3	44
4	13
5	8
6	2
7	4
9	1
11	1
Time of pregnancy loss	
Only first trimester	92
Only second trimester	2
First and second trimester	19
First and third trimester	10
First, second and third trimester	4
Familiar history of recurrent pregnancy loss	
Yes	24
No	92
No data	11

In total, we evaluated 127 karyotypes of female partners and 98 karyotypes of male partners. An abnormal karyotype of at least one partner was detected in 10 couples (7.8%). The group with chromosome abnormalities was older than the group with normal karyotype (female age 38.3 ± 3.7 vs. 32.8 ± 5.7 years, p = 0.003 (Table 1). The mean number of miscarriages was. 3.3 ± 1.1 in couples with chromosome abnormalities and 3.1 ± 1.5 in couples without chromosome abnormalities (Table 1). All patients with parental aberrations were offered genetic counseling.

**Table 1:** Comparison of the characteristics of couples with or without chromosome abnormalities

Comparasion	Chromosome abnormalities	No chromosome abnormalities	p-value
Number of couples	10	117	
Number of miscarriages	3.3 ± 1.1	3.1 ± 1.5	0.681
2 miscarriages	2/10 (20%)	52 (44%)	0.188
≥ 3 miscarriages	8/10 (80%)	65/117 (56%)	
Female age (years)	38.3 ± 3.7	32.8 ± 5.7	0.003
Male age (years)	39.3 ± 4.3	34.9 ±7 .4	0.083
Familiar history of RPL	3/10 (30%)	21/106 (20%)	0.725

RPL - Recurrent Pregnancy Loss

The 10 cases with chromosomal abnormalities are described individually in chart 2. Among those, 4 cases (40%) showed structural aberrations and 6 cases (60%) had a numerical anomaly.

**Chart 2:** Couples with Karyotype alterations: description of cytogenetic findings, number of previous abortions and maternal/paternal age

No	Number of previous abortions	Female Age	Female Karyotype	Male age	Male Karyotype
1	5	46	45,X[03]/46,XX[27]	33	46,XY
2	2	36	45,X[02]/46,XX[48]	No data	46,XY
3	3	37	45,X[03]/46,XX[47]	35	46,XY,qh-
4	3	42	45,X[02]/46,XX[48]	42	46,XY
5	3	40	47,XXX[02]/46XX[48]	40	46,XY
6	4	35	46,XX,t(10;13)(q11.2;q14)	34	No data
7	3	34	46,XX,t(6;16)(q25;p13.1)	43	46,XY,qh-
8	5	39	45,XX,i(13)(q10)[20]	45	46,XY
9	2	35	46,XX	40	46,XY,t(16;18)(q12.2;p11.3)
10	3	39	46,XX	40	47,XXY[01]/46,XY[49]

## Discussion

The prevalence of parental chromosomal alterations in our study was higher (7.8%) than most series described in the literature (2%–5%).^(15,16,18,20)^ but similar to that described in some tertiary referral centers.^(6,19)^ The prevalence of chromosomal abnormalities was higher among females (6.3%) compared to males (2.0%), but this difference was not statistically significant. Some studies have reported this difference while others have not.^(6,18,19,21)^ This imbalance happen because some chromosomal anomalies that are still compatible with fertility in females may cause sterility in males.^(19)^ Males with chromosomal aberrations were suggested to have lower fertility rate due to poor spermatic motility, abnormal seminal profile with azoospermia or severe oligoasthenoteratozoospermia.^(19)^

In our study, numerical chromosomal anomalies were more frequent than structural anomalies, contrasting with literature data. A single-center retrospective cohort study found karyotype abnormality rate of 3.74% in couples with RPL, being the structural abnormalities found in 3.12% of couples and abnormal numbers in 0.62% of them. Structurally abnormal cases included balanced translocation (38.02%), inversions (34.71%), Robertsonian translocation (10.74%), and numerical chromosome aberrations (16.53%).^(18)^ This difference may be explained by the limited number of altered karyotypes in our sample, reducing the precision of our prevalence estimates. Furthermore, most of our numerical chromosome aberrations were due to Turner mosaicism, which can be related to older patients.

In the present study, women with changes in their karyotype were older than normal counterparts, and 40% of them had low-grade Turner mosaicism. Mosaic aneuploidy of a sex chromosome can result from either genuine mosaicism, a technical artefact, or age-related loss.^(22,23)^ Therefore, the presence of cells with X chromosome aneuploidy should be considered as a spectrum that extends from phenotypically normal women to those who present one or more symptoms of the known conditions of X chromosome aneuploidy.^(22)^ This is corroborated by a positive correlation between X chromosome loss (XCL) and advancing age in women, with the frequency of X chromosome loss ranging from 0.07% at age <16 years to 7.3% at >65 years.^(23)^Mosaic Turner syndrome and various other forms of mosaicism frequently result in spontaneous pregnancies.^(24)^ However, the ocurrence of spontaneous abortion in patients with Turner mosaicism varied in different studies, ranging from 25% to 30%.^(25,26)^ Mechanisms, such as fetal chromosomal abnormalities or poor oocyte quality, could explain the higher rates of miscarriage in these patients.^(25)^ Miscarriages in these women were less frequent after oocyte donation.^(26)^ Women who presented low-grade Turner mosaicism in our study were all advised about the increased risk of miscarriage; and that the presence of low-grade Turner mosaicism in their karyotype may be related to aging.

It is also known that couples with balanced reciprocal translocation face a 50% risk of RPL and a 20% chance of their children having an abnormal genetic constitution.^(27)^ This is due to the potential for mispairing of translocated chromosomes during the initial meiotic division, leading to various forms of segregation and resulting in aneuploidy in gametes with translocated chromosomes.^(28)^However, no quantification of the risk associated with the translocation (6,16)(q25;p13.1) was found in the literature, and the patient with this translocation in our study, after receiving genetic counseling, became spontaneously pregnant and had a full-term delivery of an apparently healthy newborn.

Pericentric inversions are also associated with RPL. In a person with pericentric inversion, crossing over during meiotic division in their gametes may result in deletion or duplication of a segment in the involved chromosome. The mixture of monosomic and trisomic regions in a chromosome leads to miscarriage, unless the regions are small.^(29)^In the case of the patient in our study, the inversion of chromosome 13, known as isochromosome 13, is associated with the risk of only forming embryos with 13 monosomy, which are incompatible with life, or 13 trisomy. In fact, all patients with genetic disorders like translocation, pericentric inversions and mosaicism received proper information about the increased risks of infertility, spontaneous abortion, and having affected children. They were all advised about the indications, risks, and benefits of in vitro fertilization (IVF) with preimplantation genetic testing for structural rearrangements (PGT-SR), specially to mitigate offspring genetic risks. They were also advised that some authors suggest that natural conception may be a good alternative for cases like theirs.

The evidence that parental chromosomal abnormalities lead to miscarriage is still unclear, since a considerable percentage of couples with chromosomal abnormalities have successfully given birth.^(6,18)^ A recent systematic review evaluated a subsequent pregnancy outcome in couples with parental abnormal chromosomal karyotypes and RPL, and found a significant difference in the first pregnancy live birth rates (LBR) for couples with RPL with abnormal vs. normal karyotypes (58.5% vs. 71.9%). A markedly decreased first pregnancy LBR was found in couples with a translocation (52.9%) but not in couples with an inversion.^(30)^ Another systematic review showed no difference in the cumulative LBR between couples with RPL with and without chromosomal alterations, despite the increased risk of a subsequent miscarriage in couples with translocations.^(18)^

A recent systematic review investigated the use of PGT-SR versus expectant management in couples with RPL with normal or abnormal karyotypes. In the abnormal karyotype group, PGT-SR compared with expectant management did not increase the accumulated LBR, despite reducing the miscarriage rate.^(30)^ This highlights some issues that must be discussed with patients considering PGT-SR: failed or canceled cycles resulting in no transferable euploid embryos, IVF-related complications, and high cost, although it reduces the risk of having another abortion. Expectant management has clinical advantages and lower cost,^(30)^ so it can also be a safe way of reaching pregnancy.

This study has some limitations that should be considered when interpreting the results. The sample size had limited statistical power to estimate the real prevalence of chromosomal abnormalities in this population. The number of metaphases screened systematically in all cytogenetic examinations was relatively small, although it was enlarged to 50 metaphases to confirm any alterations.

## Conclusion

The prevalence of parental chromosomal alterations in our study was higher than in most series described in the literature, suggesting that karyotyping should be part of the investigation for Brazilian couples with RPL, as identifying the genetic etiology may have implications for subsequent pregnancies. No differences were found between couples with or without karyotype alterations in our study, except for female partner’s age, which was higher in the subgroup with parental chromosome alterations. Genetic counseling must be offered for all these couples so that they can choose the best treatment for them, considering risks, cost, and success probability.
